# High fat plus high cholesterol diet lead to hepatic steatosis in zebrafish larvae: a novel model for screening anti-hepatic steatosis drugs

**DOI:** 10.1186/s12986-015-0036-z

**Published:** 2015-11-14

**Authors:** Wencong Dai, Kunyuan Wang, Xinchun Zheng, Xiaohui Chen, Wenqing Zhang, Yiyue Zhang, Jinlin Hou, Li Liu

**Affiliations:** State Key Laboratory of Organ Failure Research, Guangdong Provincial Key, Laboratory of Viral Hepatitis Research, Department of Infectious Diseases, Nanfang Hospital, Southern Medical University, Guangzhou, 510515 China; Key Laboratory of Zebrafish Modeling and Drug Screening for Human Diseases of Guangdong Higher Education Institutes, Department of Developmental Biology, School of Basic Medical Sciences, Southern Medical University, Guangzhou, 510515 China

**Keywords:** Zebrafish, Hepatic steatosis, Lipid accumulation, Drug screening

## Abstract

**Background:**

Non-alcoholic fatty liver disease (NAFLD), characterized as excessive lipid accumulation within hepatocytes, is growing in prevalence. The exploitation of effective drugs for NAFLD has been proven challenging. Herein, we aimed to establish a dietary model of hepatic steatosis using transparent zebrafish larvae in which high-throughput chemical screens could be conducted.

**Methods:**

Zebrafish larvae fed with high fat (HF) diet and high fat plus high cholesterol (HFC) diet were compared to the control. We analyzed intrahepatic lipid accumulation, biological indexes and various pathways including lipid metabolism, ER stress and inflammation. In addition, the effects of ezetimibe and simvastatin on HFC diet-induced steatosis were evaluated.

**Results:**

Zebrafish larvae fed with HF and HFC diets developed steatosis for 7 and 10 days. The incidence and degree of steatosis were more severe in HFC diet-fed larvae compared with the control and HF diet-fed larvae, suggesting that adding cholesterol to the HF diet promotes the hepatic lipid accumulation. These data were confirmed by the pathological observation. Biological indexes, free cholesterol (FC), total cholesterol (TC) and triacylglycerol (TG) were elevated in the liver of HFC diet-fed larvae compared with the control and HF diet-fed larvae. Additionally, the expression levels of endoplasmic reticulum (ER) stress and lipolytic molecules (*atf6*, *hspa5*, *hsp90b1*, *pparab*, *cpt1a* and *acox3*) were significantly up-regulated in the liver of HF and HFC diets-fed larvae compared to the control, whereas the expression of lipogenic molecules (*acaca*, *fasn*, *srebf2*, *hmgcs1* and *hmgcra*) were decreased in the liver of HF and HFC diets-fed larvae compared to the control. To validate the reliability of the HFC model and utility value for screening potential anti-steaotsis drugs, HFC-fed larvae were treated with two accepted lipid-lowing drugs (ezetimibe and simvastatin). The results showed that these drugs significantly ameliorated HFC-induced steatosis.

**Conclusion:**

Our results demonstrate that the zebrafish larvae steatosis model established and validated in this study could be used for *in vivo* steatosis studies and drug screening.

**Electronic supplementary material:**

The online version of this article (doi:10.1186/s12986-015-0036-z) contains supplementary material, which is available to authorized users.

## Introduction

Non-alcoholic fatty liver disease (NAFLD) is the most common cause of human liver dysfunction and possesses an enormous risk to human health [1]. It is estimated that about 20 %-30 % of the general population suffer from simple steatosis and the prevalence of non-alcoholic steatohepatitis (NASH) is 3 %-10 % [2]. Blocking hepatic steatosis is crucial for preventing the development of NAFLD. Although there have been improvements in the progression of hepatic steatosis to more advanced liver inflammation owing to the changes of lifestyle and the use of supplements [3, 4], the pathogenesis of NAFLD remains unclear, and therapeutic drugs are still restricted.

Rodents are the traditional model organisms used to reveal mechanisms underlying NAFLD, as well as to test the therapeutic potency of agents. There are many studies that have established NAFLD models in rodents through hepatotoxic drugs and dietary treatments, as well as genetic modification [5]. However, rodents are unfit for high-throughput drug screening because these animals produce small litters, mature slowly and incur high financial costs in terms of considerable daily expenditure and raising space owing to their relatively large body size. Furthermore, rodents must be sacrificed and their internal organs should be isolated in order to examine their liver for abnormalities. This requirement does not allow for continuous and real-time monitoring of liver condition in large samples [6]. Current situation highlights new approaches to complement rodent-based researches. Nowadays, zebrafish (*Danio rerio*) with its unique advantages in physiology, genetics and genomics, have come to be recognized as an attractive model organism for studying human disorders and screening drug candidates on a large scale [6–8]. Specific to the study of NAFLD, the livers of larvae are matured by 4 days post fertilization (dpf) and possess the main types of liver cells that are anatomically and functionally analogous to those of mammals [9, 10]. Moreover, lipid metabolism in zebrafish, which have been reported as absorption occurs in the intestine tract with the help of bile produced by the liver, cholesterol transport is mediated by lipoproteins, and triglyceride is stored in intramuscular adipocyte, subcutaneous, and visceral, are similar to humans [11, 12]. Additionally, lipid accumulation can be visualized directly in the livers of transparent larvae and chemical agents can be simply added to fish water [13–15]. These advantages make them a powerful system for studying lipid-related liver disease and assessing the effects of drugs across hundreds of fishes via a relatively simple protocol *in vivo*.

Zebrafish models of NAFLD are well established by mutagenesis or transgenic technology, chemical and dietary treatment in recent years [6, 8]. Studies have shown that zebrafish develop hepatic steatosis in response to methionine metabolism disorder [16], endoplasmic reticulum stress [17, 18] and aberrant β-hydroxybutyrate transport [19]. Hepatotoxic small molecules, such as thioacetamide, tunicamycin and γ-hexachlorocyclohexane cause an increase of hepatic lipid content [20–23]. Dietary-induced models of NAFLD in adult fish have involved forced overfeeding or high-fat diet [24–26]. Based on previous reports, there has been a lack of knowledge about diet-induced steatosis in larval zebrafish. Recently, Sapp et al. have reported that zebrafish larvae treated with fructose exhibited steatosis and inflammation, indicating that zebrafish larvae is a promising model organism to study diet-induced steatosis [27]. Along with an increase use of fructose, a growing consumption of high fat and cholesterol-enriched food, have been implicated in the rise of obesity and the prevalence of NAFLD [28, 29]. Furthermore, various evidences indicate that dietary cholesterol plays a critical role in the development of steatosis and the progression to steatohepatitis [29–31]. Herein, we fed larvae a high fat diet alone or combined with high cholesterol diet to induce steatosis and determined whether the hepatic manifestations in these fishes were similar to those of humans. In addition, ezetimibe and simvastatin were used to confirm the reliability of HFC diet-induced hepatic steatosis model in larvae. Our results indicate that the zebrafish larvae hepatic steatosis model developed in this study is convenient and low-cost for rapid assessment of anti-steatosis agents.

## Methods

### Zebrafish care and feeding

Wild-type AB stains embryos were maintained according to standard protocol [32]. Zebrafish larvae were fed with control diet (Zeigler Bros, Inc., USA. Protein: 50 %, Fat: 12 %, Fiber: 2.5 %), HF diet (Marine Biological Science Co, LTD of CNSIC, Tianjin, China. Protein: 50 %, Fat: 24 %, Fiber: 2.5 %) and HFC diet (2.5 % and 5.0 % wt/wt cholesterol added to HF diet as described [33]). This study was approved by the Animal Care and Use Committee of Southern Medical University, Guangzhou and every effort was made to minimize suffering.

### Treatments

To optimize a protocol for exposing larvae to HF and HFC diets, we first assessed the consumption of control diet by zebrafish larvae. We fed 100 larvae in a 2 L fish tank with 30 mg-180 mg per day to meet their basic energy requirement. We found that the incidence of steatosis in zebrafish larvae was gradually increased with the growing amount of feeding. Based on these results, all types of diets given to larvae were strictly restricted as 30 mg/tank per day. Ezetimibe (Santa Cruz Biotechnology, Texas, USA) used in this study was added directly to the fish tank water at concentrations of 1 μM and simvastatin (Cayman Chemicals, Michigan, USA) was added at 50 *μ*g/g food. These concentrations of drugs and methods of administration were chosen based on previous report as described [34].

### Whole-mount oil red O staining

Zebrafish larvae were fasted for 24 h after feeding. Whole larvae were then fixed in 4 % paraformaldehyde (PFA) overnight at 4 °C and washed twice with phosphate-buffered saline (PBS), infiltrated with 80 % and 100 % 1,2 propylene glycol at room temperature 20 minutes respectively and stained with 0.5 % oil red O (Cat. No.O0625, Sigma-Aldrich, St. Louis, USA) in 100 % 1,2 propylene glycol in the dark overnight at room temperature. Stained larvae were washed with PBS and faded the background color by washing with 100 % and 80 % of 1,2 propylene glycol for 30 min each and stored in 80 % 1,2 propylene glycol at 4 °C. Lipid droplets in liver tissue were observed and imaged on a bright-field dissecting microscope (Olympus szx10, Tokoyo, Japan). Larvae were defined positive for steatosis if the boundary between the liver and surrounding tissue is clear and 3 or more lipid droplets were visible within the liver by whole-mount oil red O staining.

### Oil red O staining of cryosections

Larvae were fixed in 4 % PFA overnight at 4 °C and infiltrated with 30 % sucrose overnight at 4 °C. Larvae were embedded in Tissue-Tek OCT Compound (Sakura Japan Co., Ltd., Tokyo, Japan) and 10 μm sections were mounted on slides and stored at **-** 80 °C. Before staining with oil red O, slides were warmed to room temperature and immersed in 85 % and 100 % 1,2 propylene glycol for 5 min each, and then stained with oil red O in the dark overnight at room temperature. Slides were quickly destained in 100 % and 85 % propylene glycol the following day and then washed with PBS to clean the background. Slides were visualized on an Olympus BX51 microscope.

### Biological analysis

The livers of 50-70 zebrafish larvae were homogenized in lysis buffer, centrifuged and got supernatant liquid. FC, TC and TG in the liver homogenates were measured using the kits (APPLYGEN Bioengineering, Beijing, China and Nanjing Jiancheng Bioengineering, Nanjing city, China) following the manufacturer’s instructions, and were normalized to the total protein concentration as determined by Bradford Assay (Bio-Rad, Hercules, CA).

### Histologic analysis

Larvae were fixed with 4 % PFA overnight, embedded in paraffin according to standard procedures. Four μm sections were stained with hematoxylin and eosin (H&E) and observed on BX51 microscope (Olympus, Tokyo, Japan).

### Quantitative RT-PCR

Total RNA was extracted from pools of 20-30 dissected livers and purified using RNeasy plus mini kit (Qiagen, German) and reverse-transcribed with qScript cDNA using PrimeScript™ RT-PCR Kit (Takara Biotechnology Co., Ltd., Dalian, China). Quantitative reverse-transcription PCR (qRT-PCR) was carried out on Light Cycler 480 (Roche, Basel, Switzerland) using gene-specific primers (Additional file 1: Table S1). *eef1a1* was used as a reference, and expression was calculated using the cycle threshold (Ct) method (2^-Ct (target)^/ 2^-Ct (*eef1a1*)^).

### Western blotting

Livers were dissected from pools of 20-30 larvae and lysed in RIPA buffer supplemented with protease inhibitors (Roche). The entire extract was resolved by SDS-PAGE, transferred to a PVDF membrane (Bio-Rad) and incubated overnight with antibodies as indicated. Mouse monoclonal anti-GRP78/BiP (Cat.No.MB0050, 1:1000, Bioworld Technology, St.Louis Park, USA), rabbit monoclonal anti-β-actin (Cat.No.ab151526, 1:1000, Abcam, San Franciso, USA), goat anti-rabbit-HRP (Cat.No.ab6721, 1:10000, Abcam), anti-mouse-HRP (Cat.No.ab6729, 1:10000, Abcam) were used. Quantification of band intensities was performed using Image J software.

### Statistical analyses

Statistical analysis was carried out by using SPSS 16.0 statistical software. The results are expressed as mean ± SD. Student’s t test and one-way ANOVA test are perform for analysis. Graphpad Prism 5 software (Graphpad, La Jolla, CA, USA) was used to draw figures. Differences were considered significantly at *P* < 0.05 level.

## Results

### Changes in body weight and body length in the *HF and HFC diets-fed zebrafish larvae*

**Fig. 1:**
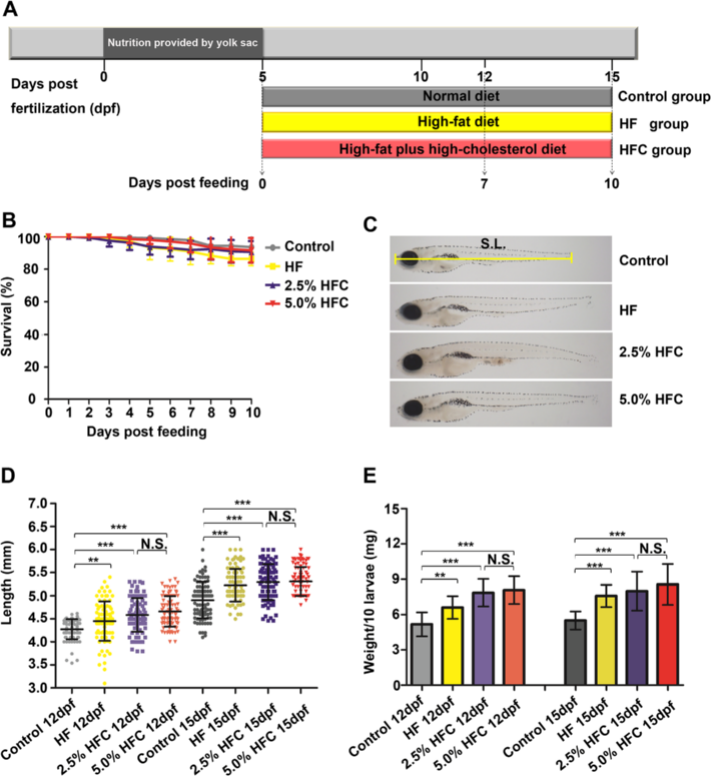
Effects of *HF and HFC* diets treatment on the survival rate and growth of zebrafish *larvae*. **a** Outline of the *HF and HFC* diets feeding protocol. **b** Larvae treated with control, HF diet, 2.5 % diet and 5.0 % HFC diet were scored for mortality, *n* = 600 from 6 tanks, error bars show standard deviation. **c** Standard length was measured from top to the end of the body. S.L.: standard length. **d** Body length and (**e**) body weight were expressed as the mean ± SD, *n* = 90 from 3 tanks. N.S.: no significant difference, **P < 0.01, ***P < 0.001, by one-way ANOVA and LSD post-hoc test

To establish a hepatic steatosis model, 5 dpf zebrafish larvae were treated with control, HF diet and two types of HFC diets (2.5 % HFC diet and 5.0 % HFC diet) for 7 or 10 days (Fig. 1a). During the period of feeding, HF diet and HFC diet did not induce significant mortality (Fig. 1b). Body length was significantly higher in the HF group and HFC groups compared to the control after 7 days feeding (*P* < 0.01, *n* = 90 all), this trend was maintained throughout the 10 days dietary protocol (Fig. 1c and d). Next, we pooled 10 larval zebrafish for each sample to measure their body weight because the weight of a single fish was too light. The control group weighed significantly less than the HF and HFC groups within 7 or 10 days feeding (*P* < 0.01, *n* = 90 all) (Fig. 1e).

### *HF and HFC* diets-induced hepatic steatosis in zebrafish larvae

**Fig. 2:**
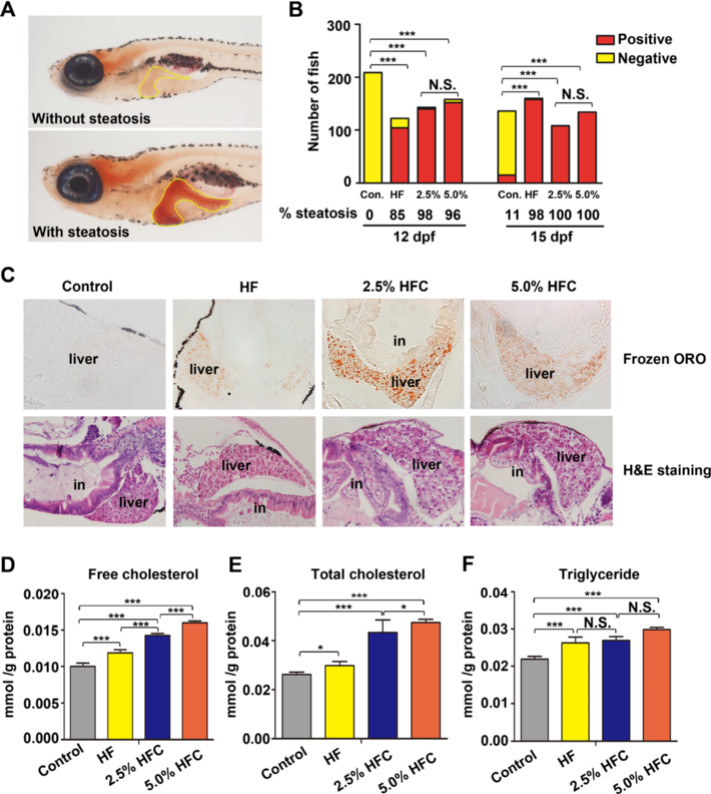
*HF and HFC* diets lead to hepatic steatosis in zebrafish larvae. **a** Representative image of larvae defined positive for steatosis by whole-mount oil red O staining. Dotted line outlines the liver. **b** The percent of larvae with steatosis was scored in 3 tanks with at least *n* = 100 per group. **c** Numberous lipid droplets in the hepatocytes were observed by oil red O staining of frozen liver sections and H&E staining in HF group, 2.5 % HFC group and 5.0 % HFC group after 10 days of feeding. **d** FC, (**e**) TC and (**f**) TG levels in livers of larvae fed with control, HF, 2.5 % HFC and 5.0 % HFC diets for 10 days were normalized to total protein. Data are expressed as mean ± SD, N.S.: no significant difference, *P < 0.05, ***P < 0.001, by one-way ANOVA. (A, ×63 magnification; C, ×400 magnification; in: intestine)

HF or HFC diets exposure results in steatosis and hepatitis in adult fish and rodents models [25, 35]. To determine whether this also occurs in larvae, zebrafish fed with HF diet and HFC diet were stained with oil red O. Larvae fed with control diet rarely developed steatosis after 7 and 10 days feeding (0 % and 11 %, respectively), whereas HF diet treatment resulted in 85 % and 98 % of steatosis. The incidence of steatosis was much higher in 2.5 % HFC group (98 % and 100 %) and 5.0 % HFC group (96 % and 100 %) compared with the control (Fig. 2a and b). Lipid droplets in the hepatocytes were further confirmed by frozen liver sections and histological analysis of larvae treated with HF and HFC diets, but not in the control (Fig. 2c and Additional file 1: Figure S1). Interestingly, we observed that larvae fed with HF diet developed mild steatosis, whereas zebrafish given HFC diet developed obvious steatosis, indicating that dietary cholesterol may be a contributing factor in the development of steatosis (Additional file 1: Figure S2). Next, we measured FC, TC and TG levels in the liver homogenates isolated from the larvae. The levels of FC, TC and TG were significantly elevated in the liver of HF group, 2.5 % HFC group and 5.0 % HFC group compared with the control group. However, there was no significant difference in the expression levels of TG between HF group and HFC group (Fig. 2d, e and f). Taken together, these results demonstrate that HF and HFC diets lead to steatosis and changes of biochemical indices in zebrafish larvae within a relatively short period of time.

### Genes changes in the livers of *HF and HFC* diets-fed zebrafish larvae

**Fig. 3:**
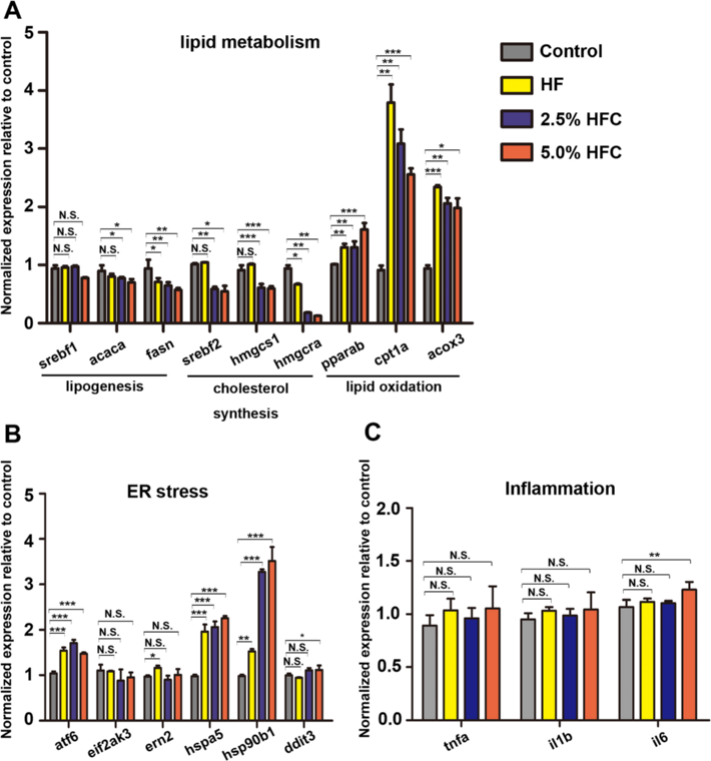
Genes changes in the livers of *HF and HFC* diets-fed zebrafish larvae. The expression levels of genes involved in (**a**) lipid metabolism, (**b**) ER stress and (**c**) inflammatory pathway in HF group, 2.5 % HFC group and 5.0 % HFC group were compared with the gene expression in control group. Gene expression analysis using cDNA prepared from pools of livers dissected from larvae (*n* =20-30) in each group after 10 days of feeding. Data are expressed as mean ± SD, N.S.: no significant difference, *P < 0.05, **P < 0.01, ***P < 0.001, by one-way ANOVA

To investigate the gene changes during *HF* and HFC diets loading period, we analyzed the mRNA expression levels of genes related to lipid metabolism, ER stress and inflammation in the liver of zebrafish larvae. The expression levels of lipogenesis genes, *acaca*, *fasn* were decreased in the livers of HF and HFC diets-fed larvae compared with the larvae in control group. The expression levels of cholesterol synthesis genes, *srebf2, hmgcs1* and *hmgcra* were consistently down-regulated in the livers of HFC diets-fed larvae compared to the control and HF diet-fed larvae. Moreover, HF and HFC diets-fed larvae have a significant increase in expression of *cpt1a* and *acox3*, as well as a rise in expression of *pparab* (Fig. 3a). In addition, the expression levels of ER stress genes, *atf6, hspa5 and hsp90b1* were elevated in the livers of HF and HFC diets-fed larvae compared to the control (Fig. 3b). Although the expression level of *il6* was elevated in the livers of 5.0 % HFC diet-fed larvae compared to the control, there were no significant differences in *tnfa*, *il1b* mRNA expression between four groups (Fig. 3c). To further study whether markers of inflammation would changes after longer duration of feeding, we analyzed the mRNA expression levels of genes related to ER stress and inflammation in the liver of the fish after 20 days of feeding. The expression levels of *atf6, hspa5, hsp90b1* and *ddit3* were consistently up-regulated in the liver of HF and HFC diets-fed larvae compared to the control. Moreover, the expression levels of inflammation genes *tnfa*, *ilb*, and *il6* were significantly elevated in the liver of HF diet and HFC diet-fed larvae compared with the control after 20 days of feeding (Additional file 1: Figure S3B and S3C). These results, along with the morphologic findings, demonstrate that HF and HFC diets treatment in larvae lead to steatosis and induce the disorder of lipid metabolism and dysfunction of ER within a relatively short period of time.

### Ezetimibe and simvastatin treatment ameliorate hepatic steatosis in 2.5 % HFC-fed zebrafish larvae

**Fig. 4:**
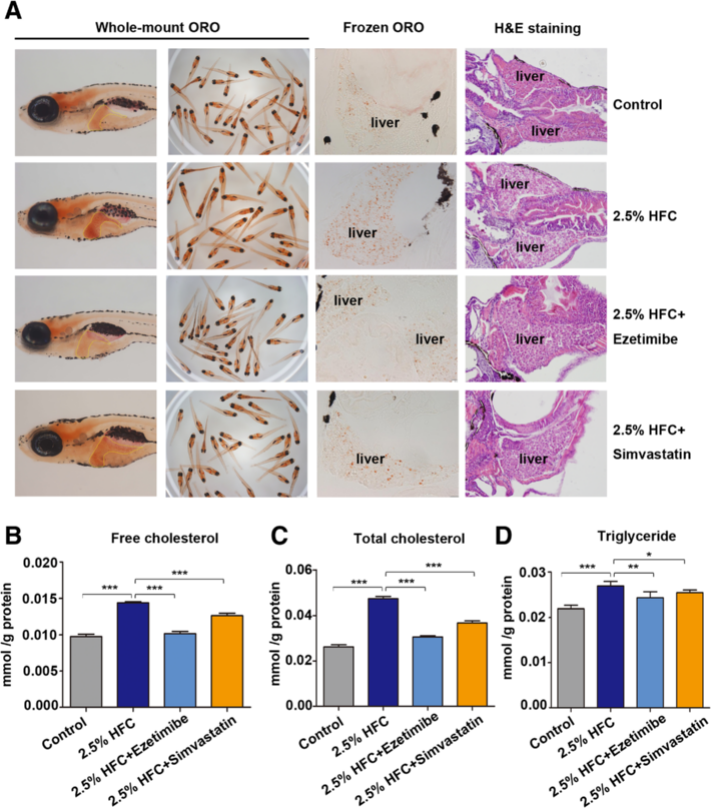
Ezetimibe and simvastatin treatment ameliorate hepatic steatosis in 2.5 % HFC-treated zebrafish larvae. **a** Whole-mount oil red O staining, oil red O staining of frozen liver sections and H&E staining of paraffin liver sections in larvae exposure to control, 2.5 % HFC, 2.5 % HFC + ezetimibe and 2.5 % HFC + simvastatin diets. Clear cytoplasmic lipid droplets were seen in livers of larvae fed with 2.5 % HFC diet. Ezetimibe and simvastatin treatment decreased lipid droplets in 2.5 % HFC-treated zebrafish larvae. **b** FC, (**c**) TC and (**d**) TG levels in livers of larvae fed with control, 2.5 % HFC, 2.5 % HFC + ezetimibe and 2.5 % HFC + simvastatin diets for 10 days were normalized to total protein. Data are expressed as mean ± SD, *P < 0.05, **P < 0.01, ***P < 0.001, by one-way ANOVA

To validate the reliability of the HFC model and utility value for screening potential anti-steaotsis drugs, HFC-fed larvae were treated with ezetimibe and simvastatin. We found that lipid droplets were decreased in the livers of 2.5 % HFC + ezetimibe and 2.5 % HFC + simavastatin groups compared with 2.5 % HFC group (Fig. 4a). Consistent with the above results, the levels of FC, TC and TG were increased in the livers of 2.5 % HFC group compared with the control group and were markedly decrease in the livers of 2.5 % HFC + ezetimibe and 2.5 % HFC + simavastatin groups (Fig. 4b, c and d). These data indicate ezetimibe and simvastatin exhibit anti-steatosis activities, which imply the potential of HFC model to test novel lipid-regulating drug candidates.

### Effect of ezetimibe and simvastatin on genes and protein expression profiles

**Fig. 5:**
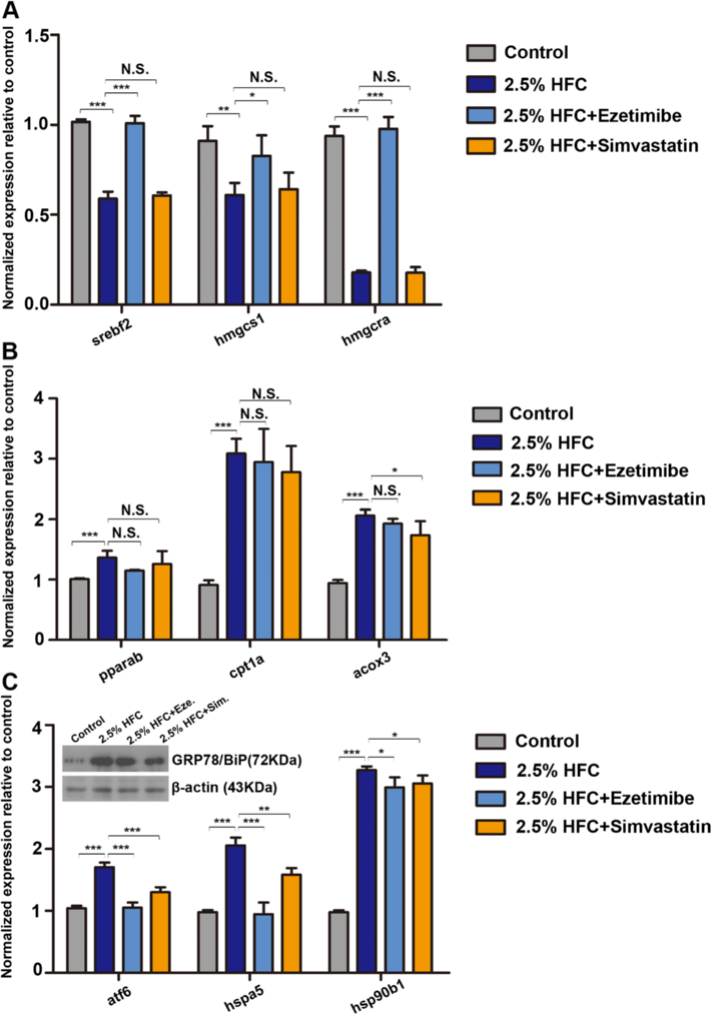
Effect of ezetimibe and simvastatin on genes and protein expression. The expression levels of (**a**) *srebf2*, *hmgcs1* and *hmgcra* (**b**) *pparab*, *cpt1a* and *acox3* (**c**) *atf6*, *hspa5 and hsp90b1* in 2.5 % HFC group, 2.5 % HFC + ezetimibe group and 2.5 % HFC + simvastatin group were compared with the gene expression in control group. Gene expression analysis using cDNA prepared from pools of livers dissected from larvae (*n* = 20-30) in each group after 10 days of feeding. Fold change in GRP78/BiP protein levels normalizing to β-actin was examined by western blot in the livers of zebrafish larvae. Data are expressed as mean ± SD, N.S.: no significant difference, *P < 0.05, **P < 0.01, ***P < 0.001, by one-way ANOVA

2.5 % HFC diet treatment significantly decreased expression of *srebf2, hmgcs1* and *hmgcra.* However, ezetimibe treatment of 2.5 % HFC-fed larvae increased mRNA expression of *srebf2, hmgcs1* and *hmgcra*, but this was not found in 2.5 % HFC + simvastatin group (Fig. 5a). Although the mRNA expression of *acox3* were decreased in the livers of 2.5 % HFC + simvastatin group compared to 2.5 % HFC group, there were no remarkable differences in expression of *pparab*, *cpt1a* in the livers of 2.5 % HFC + ezetimibe group and 2.5 % HFC + simvastatin group compared with 2.5 % HFC group (Fig. 5b). Additionally, ezetimibe and simvastatin treatment could inhibit 2.5 % HFC diet-induced ER stress and reduce the expression of *atf6*, *hspa5* and *hsp90b1* at mRNA level. Consistent with the changes of mRNA level, the protein expression of GRP78/BiP was decreased in the 2.5 % HFC + ezetimibe and 2.5 % HFC + simvastatin group (Fig. 5c). These results suggest that ezetimibe and simvastatin significantly ameliorate HFC-induced steatosis might via regulating lipid metabolism or ER stress pathway.

## Discussion

Here we have demonstrated that HF and HFC diets treatment of zebrafish larvae induced a significant phenotype similar to human steatosis, characterized by excessive lipid droplets deposit in the liver and genes expression changes involved in lipid metabolism and ER stress. Moreover, the incidence and degree of steatosis were more severe in HFC diet-fed larvae compared with the HF diet-fed larvae, suggesting that cholesterol promotes the hepatic lipid accumulation. We then confirmed that steatosis could be ameliorated by ezetimibe and simvastatin treatment, supporting the use of HFC model for testing anti-steatosis drugs. This model will be a low-cost and effective system for studying mechanisms of the pathological processes in early steatosis, as well as for testing novel lipid-regulating agents.

The advantages of zebrafish as a model organism are versatile. Symptomatic fish are generated quickly in large numbers in a single experiment, which provides distinct benefits over rodent models [6, 8]. Previous studies have proved their utility in modeling NAFLD induced by all kinds of mechanisms, but there has been a lack of a tractable model to study diet-induced steatosis in larval zebrafish [16–26]. Coincidentally, Sapp et al. have recently established a fructose-induced NASH model in 7 dpf larvae, our HFC model of steatosis therefore would be an indispensable complement for fructose model to understand diet-induced steatosis in larval zebrafish [27]. In contrast, our HFC model of steatosis induction was superior and the background steatosis was inferior to fructose model. This might because the food intake was strictly restricted every day and the raising period was relatively longer. Moreover, we observed that adding cholesterol to HF diet promotes the hepatic lipid accumulation by whole-mount oil red O staining. Consistent with the staining results, hepatic FC and TC levels in HFC group were much higher than the control and HF groups. These findings, along with previous studies, demonstrates that dietary cholesterol is a critical factor in the development of steatosis and FC accumulated in the liver is related to the severity of steatosis or liver injury in larvae as well as in mammals [29–31].

Regarding to the gene changes of zebrafish during HF and HFC diets feeding periods, we found that HF and HFC diet-fed zebrafish possessed higher mRNA levels of genes involved in lipid oxidation (*pparab*, *cpt1a* and *acox3*) and lower mRNA levels of genes involved in lipid synthesis (*acaca, fasn, srebf2, hmgcs1 and hmgcra*) than the control diet-fed ones, suggesting that excessive fat and cholesterol in diet stimulated the hepatic catabolic pathway and inhibited the anabolic pathway. Furthermore, ER stress pathway which is known to incur steatosis, promote the progression of NASH, and activate the inflammatory signaling was also affected [36, 37]. Here, we found that the expression levels of *atf6, hspa5 and hsp90b1* were elevated in the livers of HF and HFC diets-fed larvae compared to the control, indicating that HF and HFC diets lead to the dysfunction of ER. Although 10 days of HF and HFC diets exposure did not lead to a significant elevate in liver inflammation, the expression levels of *tnfa, il1b and il6* were remarkable increased in the livers of HF and HFC diets-fed larvae after 20 days of feeding. These data suggested that HF and HFC diets lead to the disorder of lipid metabolism and dysfunction of ER within a relatively short period of time. Moreover, HF and HFC diets-fed larvae have a trend toward the progression of NASH after longer duration of feeding.

The ability to conduct high-throughput drug screening is an outstanding advantage of larvae model for NAFLD studies. Unlike adult fish, lipids can be visualized directly in transparent larvae and juveniles using whole-mount oil red O staining method [13, 38–41]. This simple method provides a high-throughput means to detect the incidence of steatosis in larvae, enabling us to examine steatosis across a large number of fish. Here, we chose two drugs, ezetimibe and simvastatin, to demonstrate the validity of our model to screen for anti-steatosis drugs. Accumulating evidences indicate that ezetimibe and simvastatin can improve steatosis and NASH in fish models and mammals [42–44]. In our HFC model, we also found that these two drugs induced a significant decrease lipid accumulation in the liver, which implies the potential of this model to test novel lipid-regulating drugs.

Here, we established a larval zebrafish model of steatosis within a relatively short period maintained on diets. Our HFC diet-fed steatosis model has several advantages. First, diet induced-steatosis most closely resembles the human pathology of NAFLD. HFC diet is not a special diet and easily imitates human dietary habits. Second, zebrafish models of NAFLD in previous studies mostly focused on the stage of early larvae and adult zebrafish, there is lack of late larvae and early juveniles models of NAFLD. In consideration of this, HFC model was useful for providing more information about diet-induced steatosis in the period of late larvae and early juveniles. Third, this model has great potential for exploring the pathogenesis of early steatosis and conducting high-throughput drug screening. Nevertheless, this model has some disadvantages. For example, female fish that rarely mated with males are apt to have offspring with poor quality, which may cause high mortality. Furthermore, multiple serum biochemical parameters are hard to detect because larval zebrafish are too small to draw blood. Additionally, it’s a time-consuming work to dissect the livers from the small fish.

In conclusion, we established a novel larval zebrafish model of hepatic steatosis using HFC diet treatment. This model has great potential for exploring the relationships between diet and host factors that contribute to the pathogenesis of hepatic steatosis and the ability to conduct high-throughput drug screening is crucial for the development of new interventions or therapies. Our model will provide a powerful new tool in the search for new drugs to prevent and treat NAFLD.

## Conclusion

This study developed and validated a larval zebrafish hepatic steatosis model that could be used for *in vivo* screening and efficacy assessment of anti-steatosis drugs. This larval zebrafish hepatic steatosis model is easily available, low-cost with a short testing time and could speed up anti-steatosis drug research and development.

## Additional file

Additional file 1: Figure S1.
*HF and HFC* diets lead to hepatic steatosis in zebrafish larvae. **Figure S2.** Lipid accumulation in the livers and blood vessels of zebrafish larvae fed with HF and HFC diets. **Figure S3.** Genes changes in the livers of *HF and HFC* diets-fed zebrafish larvae. **Table S1.** Primer sequences used for quantitative RT-PCR. (DOCX 2678 kb)

## Abbreviations

NAFLD: Non-alcoholic fatty liver disease
NASH: Non-alcoholic steatohepatitis
ER stress: Endoplasmic reticulum stress
dpf: Days post fertilization
*srebf1*: Sterol regulatory element binding transcription factor 1
*srebf2*: Sterol regulatory element binding transcription factor 2
*acaca*: *Acetyl-CoA carboxylase alpha*
*fasn*: Fatty acid *synthase*
*hmgcs1*: *3-hydroxy-3-methylglutary-CoA synthase 1 (soluble)*
*hmgcr*: *3-hydroxy-3-methylglutary-CoA reductase a*
*pparab*: *Peroxisome* proliferator-activated receptor alpha b
*cpt1a*: *Carnitine palmitoyltransferase 1a*
*acox3*: *Acyl-CoA oxidase 3*
*atf6*: Activating transcription factor 6
*eif2ak3*: *Eukaryotic* translation initiation factor *2-alpha kinase 3*
*ern2*: *Endoplasmic reticulum to* nucleus signaling 2
*hspa5*: Heat shock protein 5
*hsp90b1*: Heat shock protein 90*, beta (grp94),* member 1
*ddit3*: DNA-damage-inducible transcript 3
*il1b*: *Interleukin 1, beta*
*tnfa*: *Tumor necrosis factor a* (TNF superfamily, member 2)
*eef1a1*: *Eukaryotic* translation elongation factor *1 alpha 1*
