# Supplementary material for: High fat plus high cholesterol diet lead to hepatic steatosis in zebrafish larvae: a novel model for screening anti-hepatic steatosis drugs
Source: Nutr Metab (Lond). 2015 Nov 14;12:42. doi: 10.1186/s12986-015-0036-z (PMC4650307; doi:10.1186/s12986-015-0036-z)
Supplement: Additional file 1: Figure S1. — HF and HFC diets lead to hepatic steatosis in zebrafish larvae. Figure S2. Lipid accumulation in the livers and blood vessels of zebrafish larvae fed with HF and HFC diets. Figure S3. Genes changes in the livers of HF and HFC diets-fed zebrafish larvae. Table S1. Primer sequences used for quantitative RT-PCR. (DOCX 2678 kb) [file 12986_2015_36_MOESM1_ESM.docx]

**Supplementary Material**

**
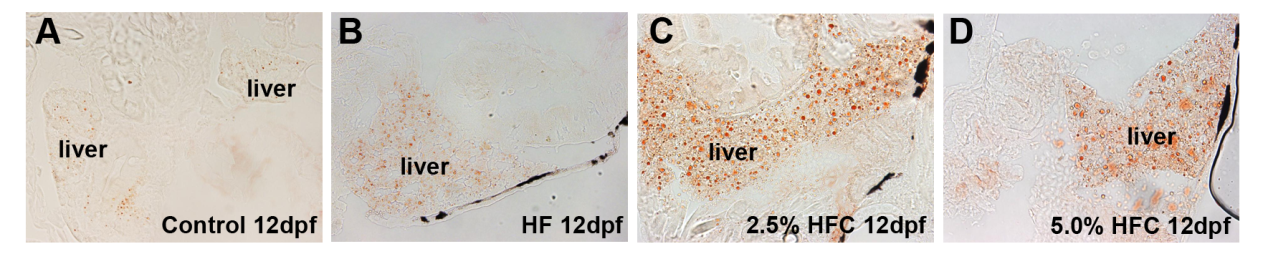
**

**Fig.S1** HF and HFC diets lead to hepatic steatosis in zebrafish larvae. Oil red O staining of frozen sections through the livers of larvae fed with control, HF, 2.5% HFC and 5.0% HFC diets for 7 days (×400 magnification). (A) Control group, normal diet-treated group; (B) HF, high fat diet-treated group; (C) 2.5% HFC, high fat plus 2.5% cholesterol diet-treated group; (D) 5.0% HFC, high fat plus 5.0% cholesterol diet-treated group.

**
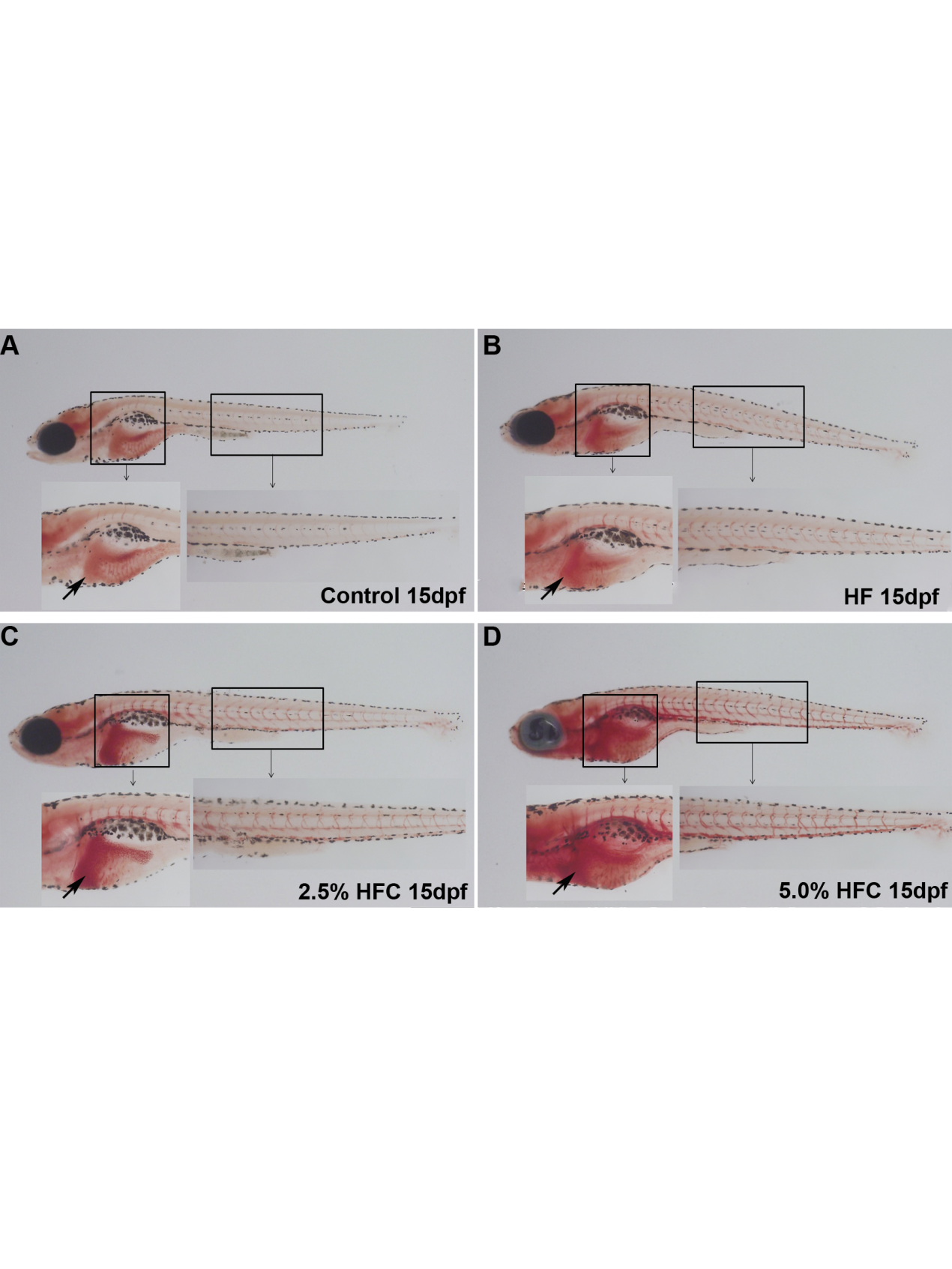
**

**Fig.S2** Lipid accumulation in the livers and blood vessels of zebrafish larvae fed with HF diet, 2.5% HFC diet and 5.0% HFC diet. Larvae of 5 dpf fed with control, HF diet, 2.5% HFC and 5.0% HFC diets, collected at 10 days and stained with oil red O. Zebrafish larvae fed with the HF diet developed mild steatosis, whereas zebrafish given the HFC diets developed obvious steatosis (×25 magnification). Black arrow indicated the liver (×63 magnification). (A) Control group, normal diet-treated group; (B) HF, high fat diet-treated group; (C) 2.5% HFC, high fat plus 2.5% cholesterol diet-treated group; (D) 5.0% HFC, high fat plus 5.0% cholesterol diet-treated group.

**
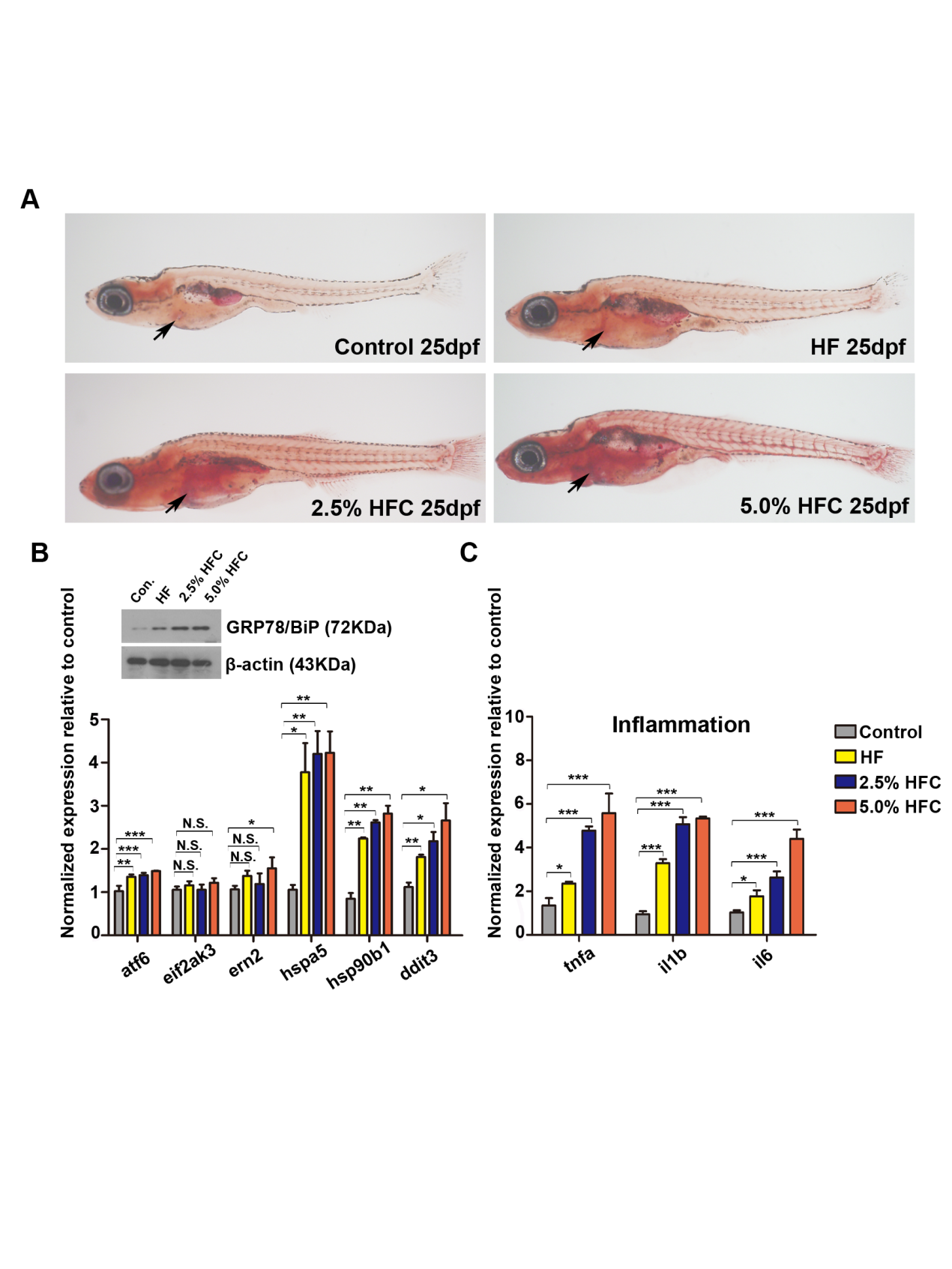
Fig.S3** **Genes changes in the livers of HF and HFC diets-fed zebrafish larvae.** (A) Larvae of 5 dpf fed with control, HF diet, 2.5% HFC and 5.0% HFC diets, collected at 20 days and stained with oil red O. The expression levels of genes involved in (B) ER stress and (C) inflammatory pathway in HF group, 2.5% HFC group and 5.0% HFC group were compared with the gene expression in control group. Gene expression analysis using cDNA prepared from pools of livers dissected from larvae (*n* =20-30) in each group. Data are expressed as mean±SD, N.S.: no significant difference, **P<*0.05, ***P<*0.01, ****P<*0.001, by one-way ANOVA.

**Table S1. Primer sequences used for quantitative RT-PCR**

| Gene | Accession | Forward Primer | Reverse Primer |
| --- | --- | --- | --- |
| *srebf1* | NM_001105129 | CATCCACATGGCTCTGAGTG | CTCATCCACAAAGAAGCGGT |
| *acaca* | NM_001271308 | GCAAGTGTGGTTCCCTGATT | TCATGAAGGTCAGCGAACTG |
| *fasn* | XM_009306806 | GAGAAAGCTTGCCAAACAGG | GAGGGTCTTGCAGGAGACAG |
| *srebf2* | NM_001089466 | CACTCACACAAGCACACACG | ACCTGGTTCTGGATGAATCG |
| *hmgcs1* | NM_201085 | CTCACTCGTGTGGACGAGAA | GATACGGGGCATCTTCTTGA |
| *hmgcra* | NM_001079977 | CTGAGGCTCTGGTGGACGTG | GCAGCTACGATGTTGGCG |
| *pparab* | NM_001102567 | CGTCGTCAGGTGTTTACGGT | AGGCACTTCTGGAATCGACA |
| *cpt1a* | XM_005166476 | ACTCTCGATGGACCCTGTGA | CTGGATGAAGGCATCTGGAC |
| *acox3* | NM_213147 | AAGGACATCGAGCGAATGAT | CTATGAAAGAGTGGAGGCCG |
| *atf6* | NM_001110519 | CTGTGGTGAAACCTCCACCT | CATGGTGACCACAGGAGATG |
| *eif2ak3(perk)* | XM_005156585 | TGGGCTCTGAAGAGTTCGAT | TGTGAGCCTTCTCCGTCTTT |
| *ern2* | XM_001919315 | TGACGTGGTGGAAGTTGGTA | ACGGATCACATTGGGATGTT |
| *hspa5(bip)* | NM_213058 | AAGAGGCCGAAGAGAAGGAC | AGCAGCAGAGCCTCGAAATA |
| *hsp90b1(grp94)* | NM_198210 | ATGGCACCAAGAAGAATGACTT | GCACCAGCTTTTTACGGATAAC |
| *ddit3(chop)* | NM_001082825 | AAGGAAAGTGCAGGAGCTGA | TCACGCTCTCCACAAGAAGA |
| *il1b* | NM_212844 | TGGCGAACGTCATCCAAG | GGAGCACTGGGCGACGCATA |
| *il6* | NM_001261449 | AGACCGCTGCCTGTCTAAAA | TTTGATGTCGTTCACCAGGA |
| *tnfa* | NM_212859 | GCTTATGAGCCATGCAGTGA | TGCCCAGTCTGTCTCCTTCT |
| *eef1a1* | NM_131263 | TACTTCTCAGGCTGACTGTG | ATCTTCTTGATGTATGCGCT |
